# Aluminum induces rapidly mitochondria-dependent programmed cell death in Al-sensitive peanut root tips

**DOI:** 10.1186/s40529-014-0067-1

**Published:** 2014-09-03

**Authors:** Wen-Jing Huang, Thet Lwin Oo, Hu-Yi He, Ai-Qin Wang, Jie Zhan, Chuang-Zhen Li, Shan-Qing Wei, Long-Fei He

**Affiliations:** College of Agronomy, Guangxi University, Daxue Road 100, Nanning, 530004 PR China

**Keywords:** Al toxicity, Programmed cell death, Arachis hypoganea L, Caspase3-like, Mitochondria, Reactive oxygen species burst

## Abstract

**Background:**

Although many studies suggested that aluminum (Al) induced programmed cell death (PCD) in plants, the mechanism of Al-induced PCD and its effects in Al tolerance is limited. This study was to investigate the mechanism and type of Al induced PCD and the relationship between PCD and Al tolerance.

**Results:**

In this study, two genotypes of peanut 99-1507 (Al tolerant) and ZH2 (Al sensitive) were used to investigate Al-induced PCD. Peanut root growth inhibition induced by AlCl_3_ was concentration and time-dependent in two peanut varieties. AlCl_3_ at 100 μM could induce rapidly peanut root tip PCD involved in DNA cleavage, typical apoptotic chromatin condensation staining with DAPI, apoptosis related gene *Hrs203j* expression and cytochrome C (Cyt *c*) release from mitochondria to cytosol. Caspase3-like protease was activated by Al; it was higher in ZH2 than in 99-1507. Al increased the opening of mitochondrial permeability transition pore (MPTP), decreased inner membrane potential (ΔΨ_m_) of mitochondria. Compared with the control, Al stress increased O_2_^•-^ and H_2_O_2_ production in mitochondria. Reactive oxygen species (ROS) burst was produced at Al treatment for 4 h.

**Conclusions:**

Al-induced PCD is earlier and faster in Al-sensitive peanut cultivar than in Al-tolerant cultivar. There is a negative relationship between PCD and Al resistance. Mitochondria- dependence PCD was induced by Al and ROS was involved in this process. The mechanism can be explained by the model of acceleration of senescence under Al stress.

**Electronic supplementary material:**

The online version of this article (doi:10.1186/s40529-014-0067-1) contains supplementary material, which is available to authorized users.

## Background

Aluminum (Al) is the most abundant metal in the earth’s crust. Al presents in various forms including Al^3+^, Al(OH)^+^ and Al(OH)^2+^ in soil solution as soil pH drops below 5, and these are harmful to crops. Therefore, Al toxicity is considered as a major limiting factor for crop production and quality in acid soil, which comprises 30-40% of the world’s arable lands (Pan et al. [2001]). Al affects root growth through acting in the root apical zone, resulting in growth inhibition in a very short time at micro molar concentrations (Panda and Matsumoto [2007]). Plant species have evolved diverse mechanisms of Al tolerance, including the secretion of Al-induced organic acids, immobilization of Al at cell wall, and increasing in rhizosphere pH (He et al. [2012]).

Programmed cell death (PCD) known as apoptosis, has been described in animal cells in detailed at morphological, biochemical, and genetic levels. Similar to animal cells, plant cells can also respond to various stimuli, including biotic and abiotic stresses as well as development by initiating PCD (Wang et al. [2011]). PCD is associated with specific morphological and biochemical features such as nuclear DNA degradation (Achary and Panda [2010]; Fu et al. [2003]; Qiao et al. [2003]), specific proteases activation (Poor et al. [2013]), plasma membrane embolus or chromatin condensation (Wang et al. [2012]), Cyt *c* release from mitochondria to cytoplasm (Wang et al. [2010]), mitochondrial permeability transition pore (MPTP) opening and mitochondrial membrane potential collapse (Panda et al. [2008]; Toninello et al. [2000]). Increasing evidences show that Al-induced PCD plays a significant role in Al tolerance of plants, animals and yeasts (Wang et al. [2009]; Yakimova et al. [2007]; Zheng et al. [2007]). Researches have described some apoptosis-like characters upon Al treatment in plant cells, including the appearance of DNA ladder, changes in nucleus morphology, and the fragmentation of nucleus (Panda and Matsumoto [2007]). They also determined the potential roles of anti-apoptotic members in Al tolerance (Yakimova et al. [2007]).

Mitochondria play a vital role in eukaryote life. The mitochondrial pathway of apoptosis functions in response to various types of stress including Al in both animal (Toninello et al. [2000]) and plant cells (Panda et al. [2008]; Poborilova et al. [2013]; Yamamoto et al. [2002]). In most cases of mitochondria-dependent PCD, Cyt *c* is released from mitochondria dues to the opening of a MTPT and the activation of caspase protease (Kim et al. [2006]). On the other hand, mitochondria is unique organelle which carries out important oxidation-reduction reactions. It has been considered as major reactive oxygen species (ROS) producers in animal cells and plant cells without chloroplasts, such as root cells (Tiwari et al. [2002]). Excess generation of ROS is metabolically induced by abiotic and biotic stresses in plant cells (Foyer and Noctor [2000]). ROS are not only a harmful factor to plants because they react with a large variety of biomolecules, including DNA, proteins, and carbohydrates (Siddiqui et al. [2013]), but also a signaling molecule in plants which mediates various physiological and biochemical processes (Mittler et al. [2004]) including systemic acquired resistance (SAR) and hypersensitive resistance (HR) (Huang et al. [2004]), senescence and PCD (Liu and Lin [2013]; Matsumoto and Motoda [2013]; Wei et al. [2013]; Xing et al. [2013]).

As an importance oil and food crop, peanut (*Arachis hypoganea* L.) is usually planted in acid soil in the south of China, and has high Al tolerance capacity compared to other crops. The mechanism of Al toxicity and tolerance of peanut is not uncovered. Zhan et al. ([2011]) reported firstly Al induced PCD in peanut, and the cloned *AhSAG* (a senescence-associated gene) could induce or promote the occurrence of PCD in plants. Unfortunately, it should take 4 days for Al inducing PCD in the report (Zhan et al. [2013]). The type of Al-induce PCD could not be determined in peanut, and the mechanism and regulation referred to PCD have poorly understood. The aim of our present work is to certify the phenomenon and type of Al-induced PCD in peanut in a short time, and assess the behavior of mitochondria as well as the activation of caspase-3-like activity under Al treatment. Based on the results obtained from cell imaging and biochemical approaches, it can provide a new insight into the mitochondria-dependent mechanism of Al-induced PCD in peanut root tip cells.

## Methods

### Plant materials and treatment

Plant materials and treatment were prepared as described by Zhan et al. ([2008]) with slight modifications. The seeds of peanut cultivars 99-1507 (Al-tolerant) and ZH2 (Al-sensitive) were germinated in moistened sand for 5 days in the dark at 26 C. Three cm-length root seeds were transferred in the 1/5 Hoagland nutrient solution which was exchanged at an intervals of 1 day. After the emergence of the fourth leaf, the seedlings were pretreated with 0.1 mM CaCl_2_ (pH 4.5) solution for 24 h. Some seedlings were treated respectively with different AlCl_3_ concentrations (0, 20, 50, 100, 200 and 400 μM) (pH 4.5) followed by 0.1 mM CaCl_2_ for 24 h, and pH was adjusted by HCl. The others were treated respectively with 100 μM AlCl_3_ concentrations (pH 4.5) at different times (0, 4, 8 and 12 h) followed by 0.1 mM CaCl_2_. The plants were grown in a self-regulating culture room with a 12 h/26°C day and a 12 h/20°C night cycle.

### Assay of relative root elongation

Effects of Al on different peanut cultivars root growth were observed by measuring the main root length. The relative root elongation (RRE) was calculated through the formula: RRE = L_24h_-L_0h_**/** L_24h_.

### Light microscopy (LM) observation

Tissue sections were prepared by Gladish et al. ([2006]) with some modifications. Tip segments at 5-10 mm long (n = 5-10 for each treatment) were excised and treated in osmotically balanced FAA fixative solution buffer (pH 7.2) at least 12 h. Then, the segments were rinsed in ddH_2_O three times and dehydrated through a graded series of ethanol (40, 50, 65, 80, 90, 100, 100%, 20 min for each step).

Dehydrated root tip segments were embedded in Steedman’s Wax (Polyethylene glycol 400 distearate : 1-hexadecanol = 9 : 1, w/w) as described by He et al. ([2004]) via a graded series Steedman’s Wax : ethanol-1 : 1, 2 : 1, 1 : 0, 2 h each step. Embedded root segments were sectioned at 10 μm with a Leica RM2255 ultramicrotome (Leica Instruments GmbH, Heidelberg, Germany) and mounted on glass slides. Nine or ten embedded root segments were selected at random and sectioned for each treatment. Some sections were stained in solution buffer with 0.1% hematoxylin and 0.01% KIO_3_ (pH 7.2), and observed via bright-field LM for Al absorption analysis. Other sections were stained with 1 mg L^-1^ DAPI (4’, 6-diamidino-2-phenylindol dihydrochloride) in ddH_2_O for 5 min, observed and visualized on 340-380 nm excitation with a Leica DM4000B fluorescence microscope in order to analyze nuclear changes and count PCD rate after Al treatment. The PCD rate was calculated with the percentage of apoptosis cells to total cells. Apoptosis cells morphological changes include chromatic agglutination, karyopyknosis, and nuclear fragmentation in some cells under microscope. More than 50 random fields (400×) were observed and calculated respectively by three individuals.

### Determination of Al Content

One-week-old seedlings were treated with 100 μM AlCl_3_ (pH 4.5) for different times (0, 4, 8 and 12 h), followed by 0.1 mM CaCl_2_, which were used for determination of Al content (Barry and Adam [1984]; He and Liang [1996]). At first, the sample was washed twice with distilled water, cut apical (±10 mm section) from the root tip, weighed, and soaked in 1.5 mL of 2 mM HNO_3_ solution for 24 h. The soaking solution was transferred into 25 mL volumetric flask, and 1 mL 0.1 mM HNO_3_, 2 mL 5 mM CTMAB (cetyltrimethyl ammonium), 2 mL 50 mM EDTA-Zn were added, then was shaked thoroughly for 2 min. 2 mL 0.05% chromazurol-S chromogenic reagent, 4 mL 40% hexamethylenetetramine solution was added in proper order. Finally, the flask was filled using distilled water, shaked well and took 30°C for 20 min. Meanwhile, Al standard solution was prepared for the standard curve. Absorbance was measured at 635 nm and calculated the content of Al by spectrophotometer (VIS-723).

### DNA fragmentation

After Al treatments, peanut roots of 99-1507 and ZH2 were firstly washed in running tap water, and then washed twice with ddH_2_O thoroughly. Each root was cut in 10 mm section from the root tip, weighed 0.3 g, and was homogenized in liquid nitrogen. DNAs were extracted by the CTAB protocol (Wang et al. [2009]). DNA samples were digested with 10 μL 100 μgmL^-1^DNase-free RNase and 20 μL 10 mg L^-1^ Proteinase K for 1 h at 37 C, then resuspended in 50 μL of TE buffer. Fifty μL DNA samples were loaded into 6 × loading buffer with nucleic acid dye GelRed. DNA fragments were checked on agarose gel (2%) electrophoresis at 50 V for 30 min. Results were photographed under UV excitation.

### Expression analysis of Hsr203j

The expression of *Hsr203j* in root tip cells was analyzed by semi-quantitative RT-PCR (Ma et al. [2010]). Samples were harvested separately at 0, 4, 8 and 12 h after 100 μM AlCl_3_ treatment. Total RNA was extracted with Trizol reagent protocol (Catalog No. 15596-018; Invitrogen). cDNA was synthesized from total RNA with M-MLV Reverse Transcriptase kit (Catalog No. 1621; Fermentas). The specific primers for *Hsr203j* were 5’-TTTGAGACTTGCCTTACCTG-3’ and 5’-TTACTTACCCGGAGAT TACA-3’, were designed according to the published cDNA (GenBank accession No.GU827198). The primers of positive control *actin* were 5’-ACCTTCTACAACGAGCTTCGTGTG-3’ and 5’-GAAAGAACAGCCTGAATGGCAAC-3’ (Zhan et al. [2011]). Each PCR reaction was carried out in final volume of 25 μL, containing 10 ng of first-strand cDNA, 2.5 mM MgCl_2_, 10 μM PCR primers, 2 mM dNTP and 0.5 U Taq DNA polymerase in 10 × PCR buffer. All reactions were run for 28 cycles, each consisting of 30 s at 94°C, 30 s at 56°C, 45 s at 72°C, with an initial activation step of 5 min at 94°C and an extension step of 10 min at 72°C. The PCR products were checked on agarose gels (1%) by electrophoresis.

### Measurements of caspase3-like protease activity

Cytosolic fractions from Al-treated root tip cells were used to assay caspase3-like protease (Wang et al. [2010]), according to the manufacturer’s instruction (Caspase-3 Activity Assay Kit, `Beyotime, China). Protein concentration was determined by Bradford’s method ([1976]). Equal amounts of total protein extracts (15 μg) were incubated for 12 h at 37°C with synthetic tetrapeptide DEVD-p-nitroaniline(pNA), and the addition of the substrate resulted in a signal caused by the caspase3-dependent cleavage of the chromophorepNA from the labeled substrate. Caspase3-like activity was measured at 405 nm by microplate reader iMaker (BIO-RAD, USA). Enzymatic activity was expressed as absorbance at 405 nm. Each measurement was carried out with three independent experiments.

### Separation of mitochondria from peanut root tip cells

Mitochondria were isolated by the method of Panda et al. ([2008]) with some modifications. Peanut roots treated by 100 μM AlCl_3_ for 0, 4, 8 and 12 h were washed separately in distilled water. Then, the roots were cut about 3 g, put into the mortar, and grinded in the presence of mitochondrial extract 5 mL (0.4 M sucrose, 50 mM pH 7.4 Tris-HCl buffer, 1 mM EDTA) on ice-bath. The homogenates were centrifuged at 1500 × g for 15 min and supernatants were centrifuged at 14 000 × g for 15 min. The mitochondrial precipitates were washed 3 times with the mitochondrial suspension buffer (except EDTA, the other with the preparation liquid). The final pellets containing mitochondria were made for an appropriate volume with suspension buffer and used immediately for all experimental purposes without storage. Suspensions stained with 0.02% Janus Green B were tested by oil lens of microscope to verify the quality of mitochondria. Protein concentration was determined by Bradford’s method ([1976]).

### SDS-PAGE and detection of Cyt *c* release

Twenty μg mitochondrial proteins were separated in a 12% (w/v) SDS-PAGE. After electrophoresis, the separated proteins were transferred into nitrocellulose membrane (Catalog No. FFN06, Beyotime, China), and were wetted with blocking buffer (Catalog No.P0023B, Beyotime, China) at least 1 h. After three times washing by washing buffer (Catalog No. P0023C, Beyotime, China) for 5-min each time, cytochrome *c* was probed with a primary antibody against mouse cytochrome *c* (1: 200 dilution, Catalog No. AC909, Beyotime, China) overnight. After three vigorous washings in the same washing buffer for 15 min, each membrane was incubated with a goat anti-mouse conjugated with horseradish peroxidase conjugate (1: 1000 dilution, Catalog No. A0216, Beyotime, China) at room temperature for 2 h. After several washes with washing buffer, the membrane was transferred to DAB assay reagents (Catalog No. P0203, Beyotime, China) for chromogenic detection.

### Measurement of mitochondrial membrane permeability

Isolated mitochondria were suspended with 0.2% (w/v) BSA, and the concentration of mitochondrial proteins was adjusted approximately 0.3 mg mL^-1^. For mitochondrial membrane permeability detection, the absorbance at 540 nm was determined with a spectrophotometer (Zhan et al. [2009]; Zhang and Xing [2008]).

### Measurement of mitochondrial inner membrane potential (ΔΨ_m_)

Mitochondria (0.1 mg protein mL^-1^) were incubated in a buffer (220 mM sucrose, 68 mM mannitol, 10 mM KCl, 5 mM KH_2_PO_4_, 2 mM MgCl_2_, 500 μM EGTA, 5 mM succinate, 2 μM rotenone and 10 mM HEPES, pH 7.2) supplemented with 10 μg mL^-1^ rhodamine 123(Rh-123) for 5 min. ΔΨ_m_-dependent quenching of Rh-123 fluorescence (excitation 490 nm, emission 535 nm) was measured continuously in a spectrofluorometer (Braidot et al. [1998]; Panda et al. [2008]).

### Detection of mitochondrial ROS (O2•−and H2O2)

The mitochondrial superoxide anion radical was quantified by the methods of Purvis ([1997]) and Panda et al. ([2008]) with purified mitochondrial suspension. NADH-dependent superoxide generation was assayed at 25°C by superoxide dismutase (SOD)-sensitive rate of oxidation of epinephrine to adrenochrome with an increase in absorbance at 480 nm with 4.0 mM^-1^ cm^-1^ as extinction coefficient.

H_2_O_2_ production in isolated mitochondria was measured by a non-enzymatic assay according to Panda et al. ([2008]). 20 μl mitochondrial suspension was added into 880 μl double-distilled water, followed by 100 μl titanium sulfate. The reaction mixture was incubated for 15 min at room temperature, and the oxidation of titanium sulfate was detected at 410 nm (UV160, Shimadzu, Japan). Absorbance was converted into H_2_O_2_ concentrations by using a H_2_O_2_ standard curve.

### Statistical analysis

The experiments were independently replicated three times and their mean values were subjected to data processing and statistical analysis with Excel2007 and SPSS12.0. Data are represented as mean ± SD. Statistical analysis was performed with the Student’s paired *t* test. Differences were considered statistically significant at * *P* < 0.05, **P < 0.01.

## Results

### Al accumulation in the root tips

**Figure 1:**
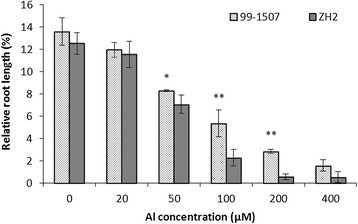
**Effects of Al concentration on relative root length.** Means and SD for relative root lengths are shown (n = 20). * and ** means significance at *P* < 0.05 and *P* < 0.01( Student’s *t* test) compared with ZH2, respectively with WT.

After AlCl_3_ (pH 4.5) stress for 24 h, low Al concentration (20 μM) could inhibit root growth significantly. Root growth of 99-1507 and ZH2 were almost arrested under 400 μM Al treatment. There were highly significant different relative root elongation rate between Al-tolerant 99-1507 and Al-sensitive ZH2 treated by Al at 50, 100 and 200 μM (Figure 1). Thus, a moderate and effective Al concentration (100 μM) was chosen to further study.

**Figure 2:**
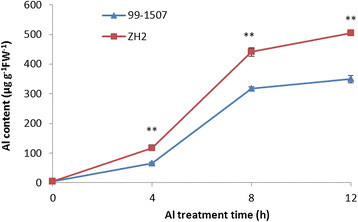
**Al content of peanut root tips treated by 100 μM AlCl**_**3**_**for 0, 4, 8 and 12 h.** * and ** means significance at *P* < 0.05 and *P* < 0.01( Student’s *t* test) compared with 99-1507, respectively.

**Figure 3:**
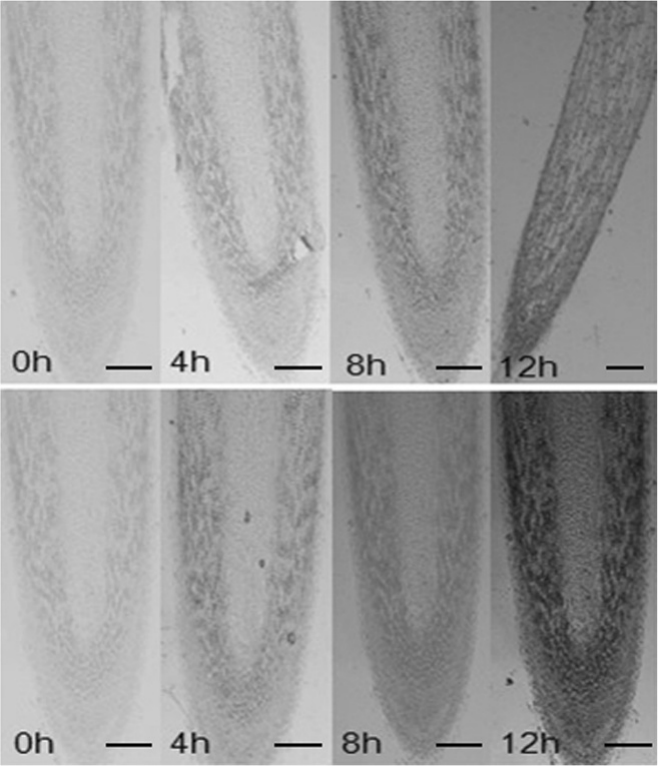
**Hematoxylin-strained images of longitudinal sections of 99-1507 (up) and ZH2 (down) treated by Al for different hours ( 50).** Scale bar indicates 200 μm.

Al contents of 99-1507 and ZH2 in peanut root tips were analyzed by spectrophotometry. Al contents increased along with exposure time extending in two peanut varieties (Figure 2), and it was higher in ZH2 than in 99-1507. Al accumulation in root tips was observed using hematoxylin staining after Al treatment. Al accumulated mainly in the epidermis and cortex of root tips. The colors of hematoxylin staining in root tip cells treated by Al for 4 h were darker compared with the control (Al treatment for 0 h). With the extension of Al treatment time, the color became deeply. Al accumulation was higher in ZH2 than in 99-1507 at the same Al stress time (Figure 3).

### Morphological changes in nuclei and DNA fragment

**Figure 4:**
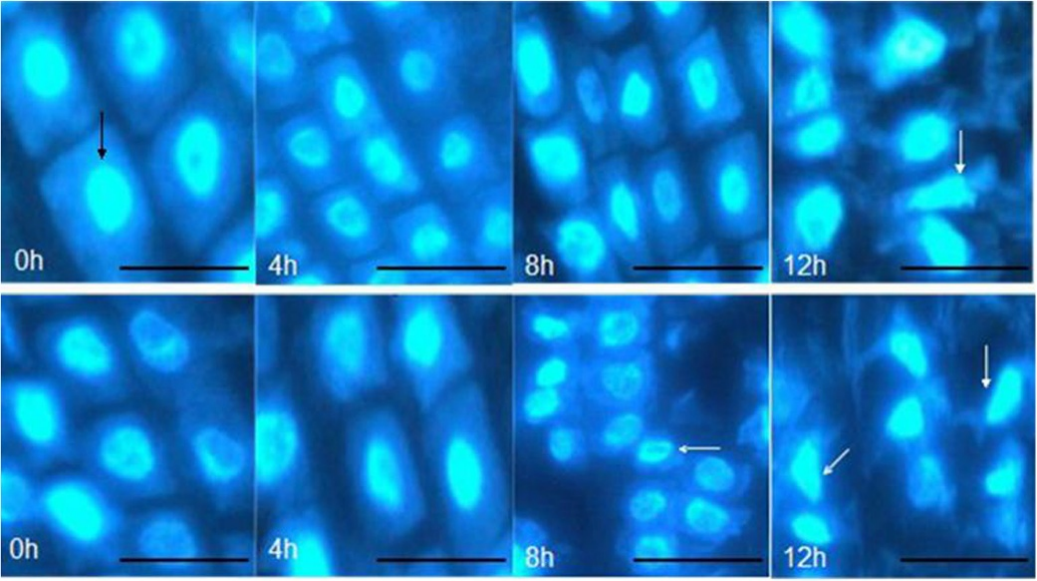
**Morphology of DAPI-stained nuclei chromatin of root tip cells in 99-1507 (up) and ZH2 (down) after 100 μM AlCl**_**3**_**treatment for different time.** Scale bar indicates 25 μm. Black arrow indicates normal cell with spherical nuclei. White arrows mean apoptotic cell undergone cytoplasmic shrinking and fragmentation with condensed and distorted nuclei.

Fluorescence microscopy clearly revealed DAPI-stained nucleus of peanut root tips under 100 μM AlCl_3_ stress for 0, 4, 8 and 12 h (Figure 4). In the cortex of primary roots of control plants (0 h), nuclei were usually spherical and located in the middle of the cell. With the increase of Al treatment time, the arrays of cell were disorder, and the integrity of cell membrane was destroyed (8 h and 12 h). Nuclei exposed to Al were flattened, lobed, invaginated or irregular in shape. The PCD ratios of root tip cells were significant difference between 99-1507 and ZH2. Treatment with 100 μM AlCl_3_ induced PCD rapidly after 4 h, while the PCD which was initiated by Al developed later in 99-1507 than in ZH2. The ratio of PCD increased significantly by Al treatment for 8 h and 12 h, and it was significant between ZH2 and 99-1507 (Table 1).

**Table 1:** **The PCD ratio of peanut root tip cells under 100 μM AlCl**_**3**_

Treatment time (h)	99-1507	ZH2
0	0%	0%
4	5 ± 2.6% *	9 ± 1.2%
8	17 ± 1.5% **	21 ± 0.8%
12	23 ± 1.8% **	30 ± 1.4%

The cell PCD ratio was calculated as a percentage of apoptosis cells to total cells in one 400 × microscopic field. More than 50 random fields were observed and calculated.

* and ** means significance at *P* < 0.05 and *P* < 0.01( Student’s *t* test) compared with ZH2, respectively.

**Figure 5:**
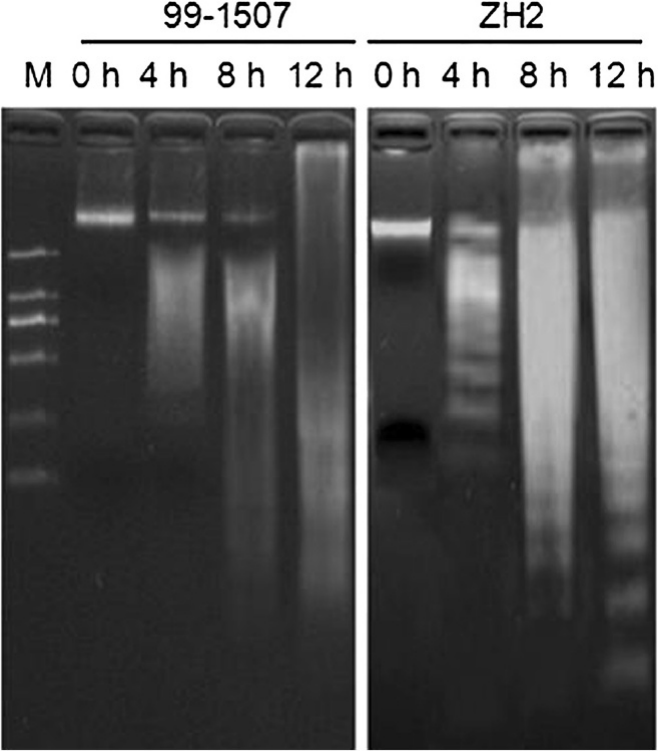
**Electrophoresis gels of DNA extracted from root tips treated by 100 μM AlCl**_**3**_ **for 0, 4, 8 and 12 h in 99-1507 and ZH2.**

DNA fragmentation was analyzed by electrophoresis on 2% agarose gel. There was no DNA ladder in 99-1507 and ZH2 under the control (100 μM AlCl_3_ for 0 h). DNA ladders were induced by Al treatment at 100 μM for 4 h in ZH2 and 99-1507, and it was very weak in the 99-1507, but it was clear in ZH2. Small DNA ladder bands increased along with the increasing of Al treatment time, and it meaned that the degree of DNA damage increased (Figure 5). In the meanwhile, it showed that the DNA damage degree of ZH2 was more distinct than that of 99-1507.

### Hsr203j expression induced by Al

**Figure 6:**
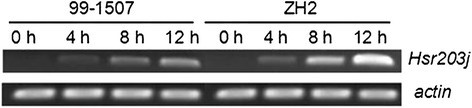
**Expression of** ***Hsr203J*** **in root tip cells treated by 100 μM AlCl**_**3**_**for 0, 4, 8 and 12 h in 99-1507 and ZH2.** The expression level of *actin* was used as a positive internal control.

It has been reported that *Hsr203* is a PCD-related gene in plants (Huang et al. [2004]; Ma et al. [2010]; Pontier et al. [2001]; Pontier et al. [1994]). To investigate the character of cell death events induced by 100 μM AlCl_3_, we analyzed the expression of *Hsr203j* in peanut root tip cells. There was not *Hsr203j* expression in root tip cells without Al treatment. Compared to the control, the expression of *Hsr203j* increased markedly after 100 μM AlCl_3_ treatment, in particular at 8 and 12 h (Figure 6). The level of *Hsr203j* expression in ZH2 was higher than in 99-1507 at the same treating time.

### Activation of caspase3-like protease

**Figure 7:**
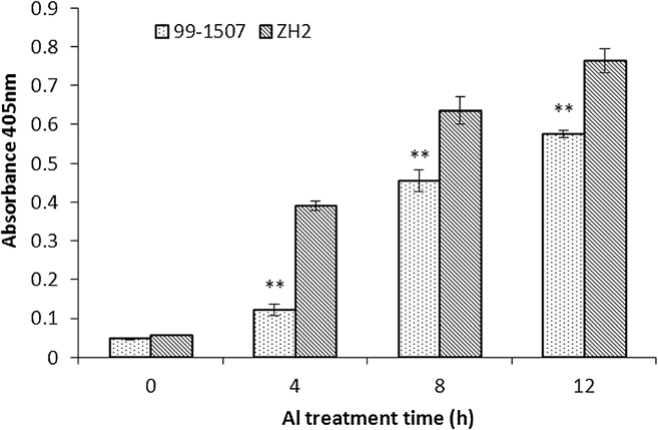
**Activities of caspase3-like protease in root tip cells treated by 100 μM AlCl**_**3**_**for 0, 4, 8 and 12 h in 99-1507 and ZH2.** Caspase3-like protease activity was expressed as the absorbance in 405 nm. Data represent the mean (±SD) of three independent measurements. ** means significance at *P* < 0.01 ( Student’s *t* test) in 99-1507 compared with ZH2.

To investigate whether caspase3-like protease was involved in in the process of Al stress-induced PCD, the activity of caspase3-like protease was analyzed. As shown in Figure 7, caspase3-like activity was in an absolutely low level without Al stress. Al treatment induced an increase in caspase3-like activity at 4, 8, and 12 h compared to the controls (0 h). What’s more, caspase3-like protease activity values of ZH2 were always higher than those of 99-1507 at Al treatment in the period of 4-12 h.

### Release of Cyt *c* from mitochondria into cytosol

**Figure 8:**
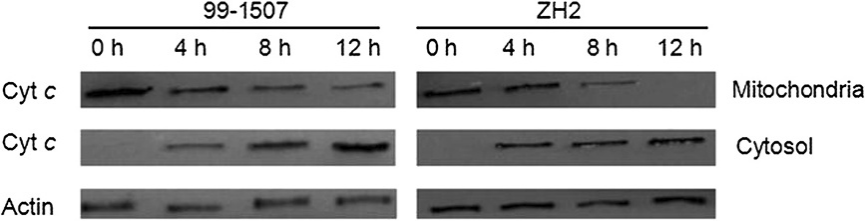
**Protein hybridization analysis of Cyt** ***c*** **in mitochondria and cytosol of peanut root tip cells treated by 100 μM AlCl**_**3**_**for 0, 4, 8 and12 h.** Equal loading was verified by anti-actin immunoblotting.

During apoptosis in animal cells, the release of Cyt *c* occurs before visible morphological changes. In order to determine whether peanut root tip cells underwent PCD during Al stress followed by the similar mitochondrial route as animal cell, we performed the immunoblotting detection of cytochrome *c* in mitochondria and cytosol. Protein hybridization showed that Cyt *c* band (about 14 kDa) was detected only in the mitochondria of control cells (0 h), but it could be detected in mitochondria and cytosol after Al stress, the bands became deep in cytosol and light in mitochondria along with the increase of Al treatment time both 99-1507 and ZH2. In the meanwhile, there was not band in mitochondria of ZH2 by Al treatment for 12 h, but there was light band in mitochondria in 99-1507 (Figure 8). The results showed that Cyt *c* released from mitochondria to cytosol after Al stress, and the speed of Cyt *c* releasing was rapider in ZH2 than in 99-1507.

### Al caused MPTP opening and ΔΨ_m_ collapse

**Figure 9:**
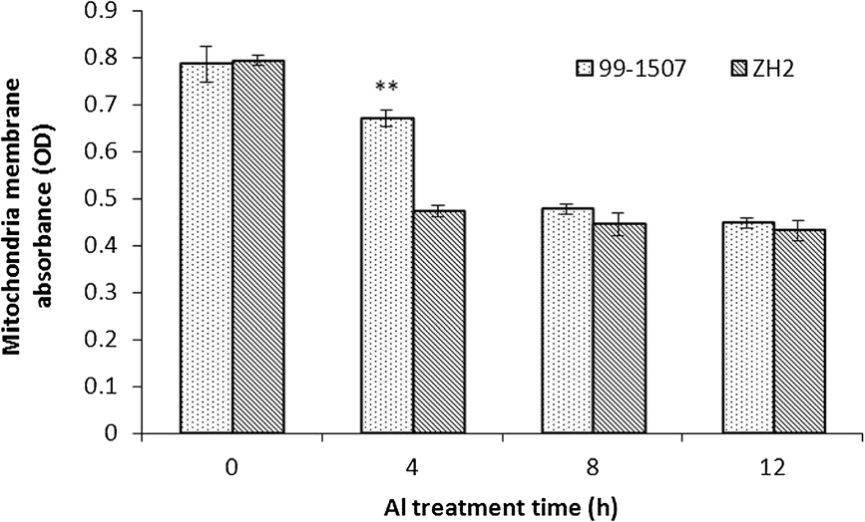
**Effects of 100 μM AlCl**_**3**_**for 0, 4, 8 and 12 h on membrane permeability of mitochondria in peanut root tip cells.** ** means significance at *P* < 0.01 ( Student’s *t* test) compared with ZH2.

When cells underwent PCD, mitochondrial membrane permeability and mitochondrial permeability transition pore opening were enhanced. Compared to the controls (0 h), mitochondrial membrane permeability decreased significantly after Al stress for 4 h, suggesting the opening of MPTP was increased by Al treatment. The mitochondrial membrane permeability was lower in ZH2 than in 99-1507 under Al stress for 4 h. It revealed that ZH2 was more sensitive to Al stress than 99-1507. There was no significant difference in mitochondrial membrane permeability between 99-1507 and ZH2 after Al treatment for 8 and 12 h (Figure 9).

**Figure 10:**
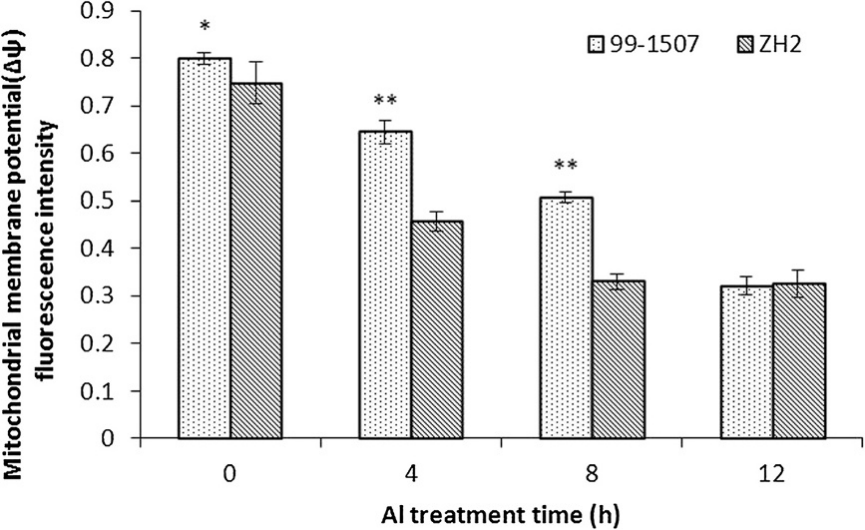
**Effects of 100 μM AlCl**_**3**_**for 0, 4, 8 and 12 h on ΔΨ**_**m**_**in peanut root tip cells.** * and ** means significance at *P* < 0.05 and *P* < 0.01( Student’s t test) compared with ZH2, respectively.

Rhodamine 123 (Rh-123) was used for monitoring the mitochondrial membrane potential shown by Emaus et al.([1986]), Panda et al. ([2008]) and Zhan et al. ([2009]) in isolated mitochondria. Under Al treatment, the mitochondria showed a time-dependent decrease in inner membrane potential compared with the control (Figure 10). After treatment with 100 μM AlCl_3_ for 4 h, the fluorescence intensity of Rh123 over all the protoplasts began to decrease. It decreased rapidly in ZH2 than in 99-1507 treated by Al for 4 h, and a further decrease was observed at 8-12 h in the 99-1507.

### Al influenced ROS production in mitochondria

**Figure 11:**
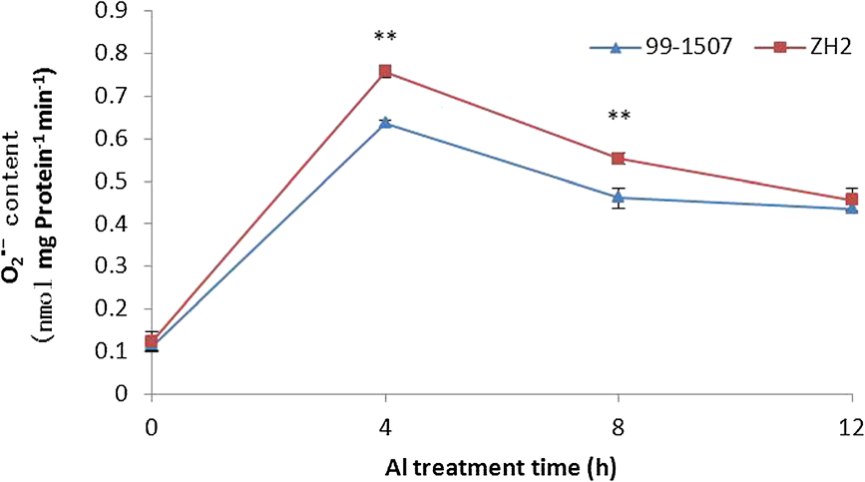
**Superoxide anion radical production of mitochondria in peanut root tip cells.** ** means significance at *P* < 0.01 ( Student’s *t* test) compared with 99-1507.

**Figure 12:**
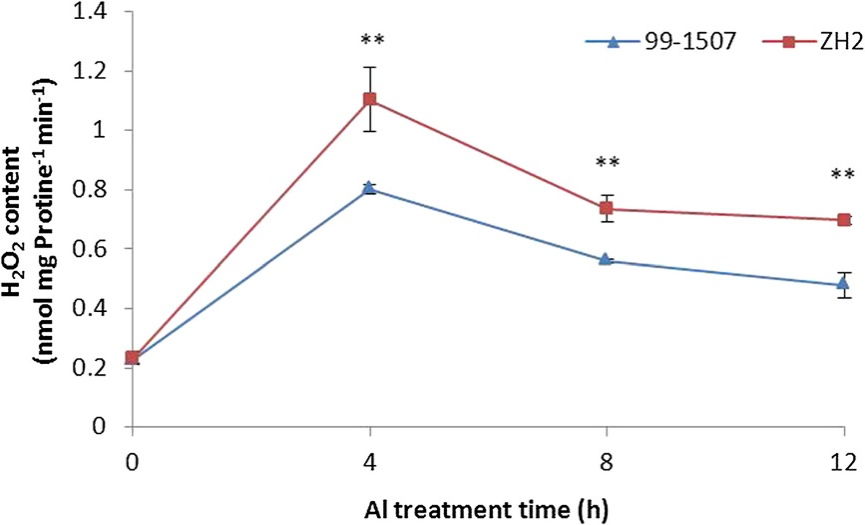
**Hydrogen peroxide production of mitochondria in peanut root tip cells.** ** means significance at *P* < 0.01 ( Student’s *t* test) compared with 99-1507.

Reactive oxygen belongs to the important markers of oxidative stress (Poborilova et al. [2013]). Whether Al-treated cells were associated with the simultaneous production of hydrogen peroxide and superoxide anion radical in mitochondria was examined. Compared to the control, Al stress increased O_2_^•-^ and H_2_O_2_ production in mitochondria from peanut root tips. The highest O_2_^•-^ and H_2_O_2_ levels in mitochondria was found after Al treatment for 4 h in 99-1507 and ZH2. The ROS production in the peanut roots which was isolated mitochondria under Al treatment increased in 0-4 h and decreased conspicuously in 8-12 h. ROS levels in Al-sensitive ZH2 were higher than in Al-tolerant 99-1507 under Al treatment (Figures 11 and 12). These results suggest that Al stress induced oxidative burst and oxidative stress in the mitochondria, and ROS participate in the Al toxicity and PCD induced by Al.

## Discussion

### Al induced rapidly PCD in root tips of peanut

Much information has been gathered on the mechanisms of Al toxicity and tolerance (Panda and Matsumoto [2007]). The initial and most dramatic symptom of Al toxicity is rapid inhibition of root elongation, and thus root relative elongation rate has served as a typical marker of levels of Al toxicity and tolerance in plants (Chandran et al. [2008]; Pan et al. [2001]; Reid et al. [1996]). Our results showed that Al stress inhibited root elongation and increased Al content, but there was difference between two varieties, which explained the high tolerance of 99-1507 to Al stress compared to ZH2 (Figures 1, 2 and 3).

The cell death in plants can be classified as PCD or necrosis. PCD is induced by abiotic stresses which includes cold (Jeong et al. [2012]), heat (Gupta et al. [2013]; Mishkind et al. [2009]), Fe^2+^ (Zhang et al. [2011]), cadmium (Li et al. [2013]), boron (Hamurcu et al. [2013]; Siddiqui et al. [2013]), salt (Lin et al. [2012]; Poor et al. [2013]; Wang et al. [2011]), ROS (Liu et al. [2012]), and hormones (Xing et al. [2013]; Zhang and Xing [2008]) in plants. Several studies found that some species of plant and suspension cultures undergo PCD after exposure to Al^3+^. Pan et al. ([2001]) reported that 0.1-50 mM AlCl_3_ induced a rapid decrease in root net growth rate, cell viability and distinct changes in cell morphology, and DNA integrity in barley. Similar results have been reported in suspension cells including tobacco (Panda et al. [2008]) and tomato (Yakimova et al. [2007]) and root tip cells of pea (Matsumoto and Motoda [2013]), but the proofs were insufficient and there are not examples in oil crops. We certified first Al induced PCD in two peanut cultivars: ZH2 (Al-sensitive) and 99-1507 (Al-tolerant) by DNA ladder, TUNEL detection and electron microscopy, and found that the concentration of Al-induced PCD was lower in ZH2 than in 99-1507 (Zhan et al. [2013]). The time of Al inducing PCD in peanut was 4 days (Zhan et al. [2013]), it is too long to research the regulation mechanism of Al induced PCD. In this study, DNA fragment were observed in root tip cells treated with 100 μM AlCl_3_ for 4 h (Figure 5). Condensation and degradation of nuclear chromatin were also observed after DAPI staining (Figure 4). These results were in agreement with the data of Fu et al. ([2003]), indicating that these hallmarks were typical not only of the animal but also of plant cell death processes during Al-induced PCD (He et al. [2012]). *Hsr203j* has been shown to be a molecular marker of the hypersensitive response (HR) (Pontier et al. [2001]). The increase of *Hsr203j* expression was taken as a hallmark of Cd induced PCD (Ma et al. [2010]). Our results showed that *Hsr203j* expression was a sensitive hallmark for Al-induced PCD in peanut. There was not the expression of *Hsr203j* in root tip cells without Al treatment, but 100 μM AlCl_3_ induced markedly the expression of *Hsr203j* especial in 8 and 12 h (Figure 6).

Taken together, these results indicate that Al triggers rapidly PCD in peanut. It certifies further that there is a negative relationship between Al-induced PCD and Al-resistance in peanut (Zhan et al. [2013]). To inhibit Al-induced PCD is one of protective mechanism to Al stress in plant.

### PCD induced rapidly by Al in peanut was mitochondria dependent

Although recent evidence has shown that Al toxicity induced cell death in plants, animals and yeasts (Fu et al. [2003]; Li et al. [2011]; Toninello et al. [2000]), the molecular mechanisms of Al-triggering evolutionarily-conserved PCD pathways have remained elusive.

Caspases are responsible for initiating, executing and signal transduction of animal PCD. Caspase-dependent as well as caspase-independent apoptosis has been described in animal cells, and the presence of caspase3-like activities is one of the peculiar biochemical features of PCD (Matsumura et al. [2000]). Animals have caspase genes; plants do not have orthologous sequences in their genomes. The existence of caspase-like proteases has been demonstrated experimentally in plants by the applications of artificial synthesized substrates and inhibitors of caspase (Li and Xing [2011]; Poor et al. [2013]; Wang et al. [2010]). Our results showed that aspase3-like protease activity was lower without Al stress; it increased markedly with Al treatment at 100 μM Al. The caspase3-like protease activity of ZH2 was higher than that of 99-1507 at different Al treatment time (Figure 7). Not only the results suggest there is caspase3-like protease in peanut root tips, but it play role in Al-induced PCD. Caspase-like proteases can be divided into three classes: metacaspases, vacuolar processing enzymes (VPEs), and saspases (Zhan et al. [2012]). Some studies showed that saspases were involved in the proteolytic degradation of ribulose-1,5-bisphosphate carboxylase/oxygenase (Rubisco) in biotic and abiotic PCD, whereas phytaspase overproducing and silenced transgenics provide evidence that phytaspase regulates PCD during both abiotic (oxidative and osmotic stresses) and biotic (virus infection) insults (Vartapetian et al. [2011]). To certify the types and the roles of caspase-like protease induced by Al in peanut root tips are next important research field.

Mitochondria play a key role in cellular metabolism and the regulation of PCD. Cyt *c* release has been certified in plant cells under-going opening of MPTP and subsequent PCD under the influence of biotic and abiotic stress conditions (Panda et al. [2008]; Tiwari et al. [2002]). Our results showed that Al increased mitochondrial membrane permeability and MPTP opening (Figure 9), decreased inner membrane potential of mitochondria (Figure 10), and released Cyt *c* from mitochondria to cytosol (Figure 8). Cyt *c* released rapidly after Al stress, which was earlier than DNA laddering and caspase3-like protease activation. It suggests that there are close relationship among mitochondrial membrane permeability, MPTP opening, inner membrane potential, Cyt *c* release, caspase3-like protease activation, and PCD production. These changes maybe occur in sequence. Mitochondria swelling enhance mitochondrial membrane permeability, result in the loss of inner membrane potential, enlarge mitochondrial permeability transition pore, and release Cyt *c*. In animals, apoptotic signals cause the loss of ΔΨ_m_ through signal transduction pathways, thereby release apoptosis-inducing factors such as Cyt *c*, consequently activate caspases, and lead cell death (Fu et al. [2003]; Kim et al. [2006]; Kroemer et al. [2007]).

A large difference between 99-1507 and ZH2 was observed in the values of MPTP, ΔΨ_m_ and Cyt *c* release after Al treatment. The absorbance value was higher in 99-1507 than in ZH2 treated by Al for 4 h; however, there were no significant difference between the control (0 h) and Al treatments for 8 and 12 h. The values of ΔΨ_m_ decreased with the increment of Al treatment time, and it was the lowest level at 8 h in Al-sensitive ZH2 and at 12 h in Al-tolerant 99-1507. It could be suggested that the stress time threshold of Al-induced PCD in Al-tolerant peanut cultivar is longer than that in Al-sensitive cultivar. That is to say, there is a negative relationship between PCD and Al resistance in peanut on Al treatment time as well as on Al treatment concentration.

It is well known that ROS act as important signal molecules in triggering PCD (Liu and Lin [2013]; Maksymiec [2007]; Matsumoto and Motoda [2013]; Xing et al. [2013]). Mitochondria have received considerable attention in the investigation of ROS-dependent PCD (Panda et al. [2008]). Many reports have shown that Al stress can induce ROS production, which is an important component of the plant’s reaction to toxic levels of Al (Li and Xing [2011]; Matsumoto and Motoda [2013]; Pan et al. [2001]; Pan et al. [2002]; Panda et al. [2008]; Xu et al. [2011]). Thus, it is very likely that Al stress indirectly initiates plant PCD through inducing ROS generation. Current evidences suggested that low dose of ROS can induce antioxidant enzymes and alleviate Al-induced oxidative stress (Xu et al. [2011]), however, when the concentration of ROS reaches a certain threshold, a signal transduction pathway which results in PCD is activated, high doses of ROS result in necrosis (Solomon et al. [1999]). In our experiments, a rapid burst of ROS formation occurred in Al-treated protoplasts for 4 h, and the ROS generated from mitochondria were demonstrated to be involved in the oxidative burst induced by Al (Figures 11 and 12). One hundred μM AlCl_3_, obviously, is an appropriate concentration to activate ROS burst leading PCD in peanut. The ROS burst provoked by Al were higher in Al-sensitive cultivar ZH2 than in Al-tolerant cultivar 99-1507. It indicated that ROS acted as signal molecules in Al-induced protoplast PCD, and the burst of ROS was an early event in this process.

In consideration of all these data, Al induced rapidly PCD in root tips of peanut, ROS of mitochondria involved in this PCD process. The PCD was mitochondria dependence, and it can be explained by the model of acceleration of senescence under Al treatments (Zhan et al. [2013]). Besides, caspase3-like protease locates between Cyt *c* and PCD. The difference between Al-tolerant and Al-sensitive genotype is that it is easier and faster to induce ROS burst by Al in Al-sensitive genotype than in Al-tolerant genotype.

## Conclusions

Al induced rapidly PCD in peanut root tip cells, which was earlier and faster in Al-sensitive peanut cultivar than in Al-tolerant cultivar. There is a negative relationship between PCD and Al resistance. Al-induced PCD was mitochondria-dependence in peanut, and ROS was involved in this process. The mechanism can be explained by the model of acceleration of senescence under Al stress.

## Authors’ contributions

LFH and AQW conceptualized the project, designed the experimental procedures, analyzed the data and revised the manuscript. WJH conducted the measure of Al content; root elongation; cell death; gene expression and detection of ROS, analyzed the data and wrote the manuscript. TLO and HYH performed the experiments regarding to the mitochondria. JZ; SQW and CZL cultured the plant. All authors read and approved the final manuscript.

## Authors’ original submitted files for images

Below are the links to the authors’ original submitted files for images. Authors’ original file for figure 1 Authors’ original file for figure 2 Authors’ original file for figure 3 Authors’ original file for figure 4 Authors’ original file for figure 5 Authors’ original file for figure 6 Authors’ original file for figure 7 Authors’ original file for figure 8 Authors’ original file for figure 9 Authors’ original file for figure 10 Authors’ original file for figure 11 Authors’ original file for figure 12

## Abbreviations

Al: Aluminum
Cyt*c*: Cytochrome C
DAPI: 4’, 6-diamidino-2-phenylindol dihydrochloride
EDTA: Ethylene diaminetetraacetic acid
EGTA: Ethylene glycol bis (2-aminoethyl) tetraacetic acid
HEPES: 4-(2-Hydroxyethyl)-1-piperazineethanesulfonic acid
LM: Light microscopy
MPTP: Mitochondrial permeability transition pores
PBS: Phosphate buffer saline
PCD: Programmed cell death
PCR: Polymerase chain reaction
ROS: Reactive oxygen species
Tris: Tris (hydroxymethyl) aminomethane
ΔΨ_m_: Mitochondria membrane potential
