# Supplementary figures and images for: Aluminum induces rapidly mitochondria-dependent programmed cell death in Al-sensitive peanut root tips
Source: Bot Stud. 2014 Sep 3;55:67. doi: 10.1186/s40529-014-0067-1 (PMC5432755; doi:10.1186/s40529-014-0067-1)

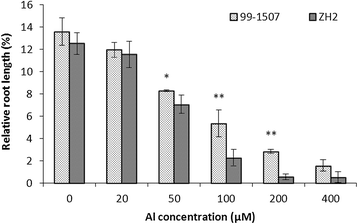

Supplement: Supplementary file 1 — Authors’ original file for figure 1 [file 40529_2014_9067_MOESM1_ESM.gif]

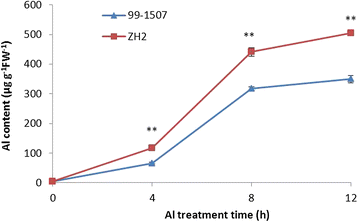

Supplement: Supplementary file 2 — Authors’ original file for figure 2 [file 40529_2014_9067_MOESM2_ESM.gif]

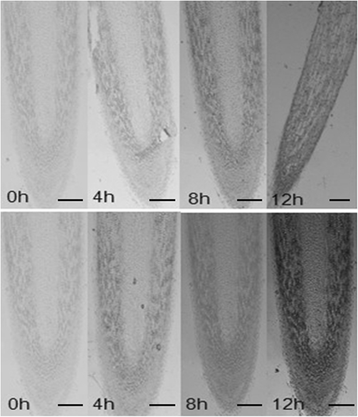

Supplement: Supplementary file 3 — Authors’ original file for figure 3 [file 40529_2014_9067_MOESM3_ESM.gif]

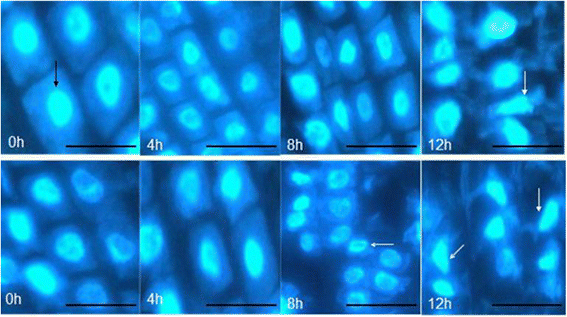

Supplement: Supplementary file 4 — Authors’ original file for figure 4 [file 40529_2014_9067_MOESM4_ESM.gif]

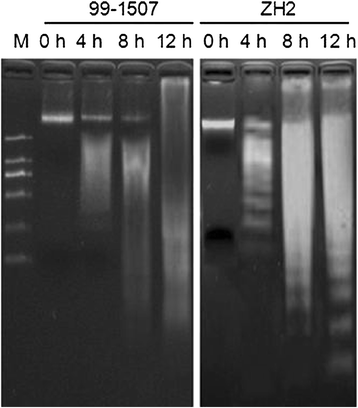

Supplement: Supplementary file 5 — Authors’ original file for figure 5 [file 40529_2014_9067_MOESM5_ESM.gif]

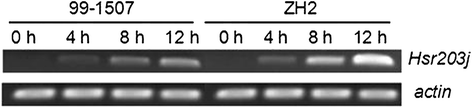

Supplement: Supplementary file 6 — Authors’ original file for figure 6 [file 40529_2014_9067_MOESM6_ESM.gif]

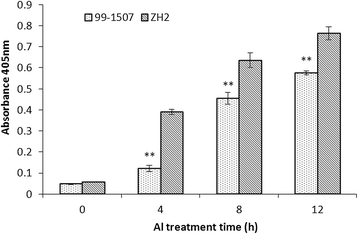

Supplement: Supplementary file 7 — Authors’ original file for figure 7 [file 40529_2014_9067_MOESM7_ESM.gif]

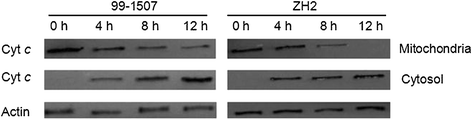

Supplement: Supplementary file 8 — Authors’ original file for figure 8 [file 40529_2014_9067_MOESM8_ESM.gif]

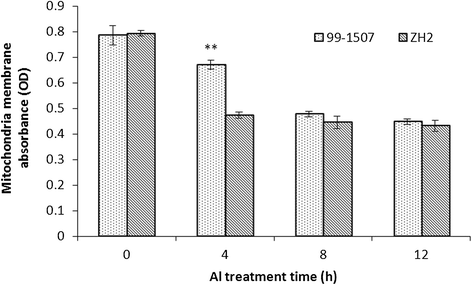

Supplement: Supplementary file 9 — Authors’ original file for figure 9 [file 40529_2014_9067_MOESM9_ESM.gif]

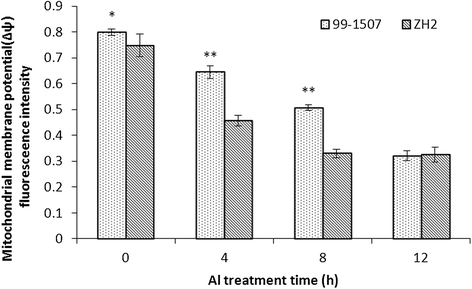

Supplement: Supplementary file 10 — Authors’ original file for figure 10 [file 40529_2014_9067_MOESM10_ESM.gif]

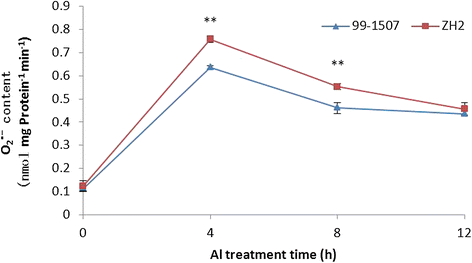

Supplement: Supplementary file 11 — Authors’ original file for figure 11 [file 40529_2014_9067_MOESM11_ESM.gif]

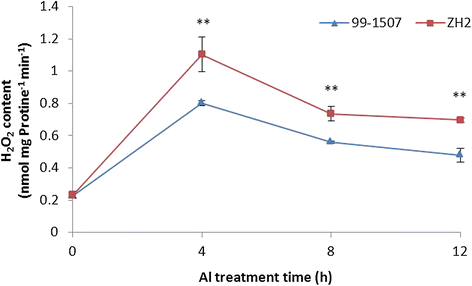

Supplement: Supplementary file 12 — Authors’ original file for figure 12 [file 40529_2014_9067_MOESM12_ESM.gif]
